# Effectiveness of treatment with pegylated interferon and ribavirin in an unselected population of patients with chronic hepatitis C: A Danish nationwide cohort study

**DOI:** 10.1186/1471-2334-11-177

**Published:** 2011-06-21

**Authors:** Nanna Hansen, Niels Obel, Peer B Christensen, Mette Kjær, Alex L Laursen, Henrik B Krarup, Axel Møller, Poul Schlichting, Jens Bukh, Nina Weis

**Affiliations:** 1 Department of Infectious Diseases, Copenhagen University Hospital, Hvidovre, 2650 Hvidovre, Denmark; 2 Department of Infectious Diseases, Copenhagen University Hospital, Rigshospitalet, 2100 Copenhagen Ø, Denmark; 3 Department of Infectious Diseases, Odense University Hospital, 5000 Odense, Denmark; 4 Department of Hepatology, Copenhagen University Hospital, Rigshospitalet, 2100 Copenhagen Ø, Denmark; 5 Department of Infectious Diseases, Aarhus University Hospital, Skejby, 8000 Aarhus, Denmark; 6 Department of Gastroenterology and Biochemistry, Unit for Molecular Diagnostics, Aalborg University Hospital, 9100 Aalborg, Denmark; 7 Department of Internal Medicine, Kolding Hospital, 6000 Kolding, Denmark; 8 Department of Gastroenterology, Herlev University Hospital, 2730 Herlev, Denmark; 9 Copenhagen Hepatitis C Program (CO-HEP), Department of Infectious Diseases and Clinical Research Centre, Copenhagen University Hospital, Hvidovre, 2650 Hvidovre, Denmark and Department of International Health, Immunology and Microbiology, Faculty of Health Sciences, University of Copenhagen, 2100 Copenhagen Ø, Denmark; 10 Faculty of Health Sciences, University of Copenhagen, Denmark, 2100 Copenhagen Ø, Denmark

## Abstract

**Background:**

The effect of peginterferon and ribavirin treatment on chronic hepatitis C virus (HCV) infection has been established in several controlled clinical studies. However, the effectiveness of treatment and predictors of treatment success in routine clinical practice remains to be established. Our aim was to estimate the effectiveness of peginterferon and ribavirin treatment in unselected HCV patients handled in routine clinical practice. The endpoint was sustained virological response (SVR), determined by the absence of HCV RNA 24 weeks after the end of treatment.

**Methods:**

We determined the proportion of SVR in a nationwide, population-based cohort of 432 patients with chronic HCV infection who were starting treatment, and analyzed the impact of known covariates on SVR by using a logistic regression analysis.

**Results:**

The majority of treated patients had genotype 1 (133 patients) and genotype 2/3 (285 patients) infections, with 44% and 72%, respectively, obtaining SVR. Other than genotype, the predictors of SVR were age ≤ 45 years at the start of treatment, completion of unmodified treatment, the absence of cirrhosis and non-European origin.

**Conclusions:**

The effectiveness of peginterferon and ribavirin treatment for chronic hepatitis C in a routine clinical practice is comparable to that observed in controlled clinical trials, with a higher SVR rate in genotype 2 and 3 patients compared to genotype 1 patients. Our data further indicate that age at start of treatment is a strong predictor of SVR irrespective of HCV genotype, with patients 45 years or younger having a higher SVR rate.

## Background

Combination therapy with peginterferon and ribavirin has improved treatment response in patients with chronic hepatitis C virus (HCV) infection, but the virus is still only eradicated in 50-80% of the patients receiving treatment [1-3], depending on HCV genotype. Treatment is lengthy and has severe side effects, which may lead to dose reduction or even prevent treatment completion. There are also several contraindications to starting treatment, such as ongoing psychiatric disease or active intravenous drug use (IDU). Progress has been made in the development of new direct-acting antiviral drugs, specifically targeting viral replication, and some of these antivirals have recently been studied in clinical trials. The results are promising especially for certain protease inhibitors expected to be licensed soon for the treatment of chronic HCV infection, as part of triple therapy with peginterferon and ribavirin [4]. It is, however, still uncertain when these new agents will be introduced into routine clinical practice.

Most previous studies of antiviral treatment response in HCV infected patients have included specific and often highly selected groups of patients (patients in clinical trials, in tertiary hospitals etc.), and these studies are therefore prone to be affected by selection bias. It was found that female gender, young age, being infected with genotype 2 or 3, absence of cirrhosis, Asian origin and early inhibition of viral replication predict a better response to treatment [5-17]. It is however, controversial whether the promising treatment effects observed in clinical trials can be transformed into an equivalent effectiveness in a routine clinical setting, although one retrospective observational cohort study by Backus et al. of patients treated in a routine clinical setting at Veterans Affairs facilities showed efficacy of treatment to be 20-52% [12].

The aim of the present study was to estimate the effectiveness of treatment for chronic HCV infection with peginterferon and ribavirin in a nationwide, population based cohort of HCV infected patients.

## Methods

### Setting

The prevalence of chronic HCV infection in Denmark was estimated by the Danish National Board of Health to be around 0.3% (15,000 individuals) [18]. Approximately 25% of these individuals are, or have been, monitored in specialized hospital departments, where antiviral therapy for HCV infection is provided for all patients found to be in need of treatment.

### The Danish Database for Hepatitis B and C (DANHEP)

DANHEP was established in January 2002 as a nationwide open cohort study with ongoing enrolment. It includes data about individuals in Denmark who are chronically infected with hepatitis B and/or C, are 16 years of age or older, are followed in one of the 11 medical departments that monitor and treat patients with chronic hepatitis in Denmark, and have been seen at least once after 1 January 2002 in one of these departments. The database is described in detail elsewhere [19].

### Tests for HCV RNA and HCV genotype

For 9 of the 11 departments recruiting patients to the DANHEP study, serum HCV-RNA was quantified in one laboratory by a previously described RT-PCR method with a detection limit of 20 IU/mL [20]. Here, HCV-genotype was determined in RT-PCR with genotype specific primers from the 5' non-coding region of the virus genome [21]. The remaining two departments in DANHEP used either COBAS AmpliPrep/COBAS TaqMan HCV test (Roche diagnostics, Branchburg, NJ) (detection limit 15 IU/mL), COBAS Amplicor HCV Monitor (Roche diagnostics) (detection limit 300 IU/mL) or qualitative HCV-RNA measurements (detection limit 50 IU/mL) to quantify HCV-RNA and the Versant HCV Genotyping Assay, LiPA (Bayer HealthCare LCC) to determine the HCV genotype. Here, genotype was also, in some cases analyzed using conserved primers deduced from the core-envelope 1-region of the virus in RT-PCR followed by sequence analysis [22]. All samples tested for the presence of HCV RNA 24 weeks after the end of treatment were tested using one of the methods with detection limit of 50 IU/mL or lower.

Although HCV genotype 6 is not found in Danish patients [22], it is widely present in Asia. We therefore re-analyzed Asian patients found to be infected with genotype 1, if the genotype was determined in RT-PCR with primers from the HCV 5' non-coding region, because this is not a valid method to distinguish genotype 1 from 6. The re-analysis was performed in RT-PCR of the Core-E1 region and the NS5B-region followed by sequence analysis of the resulting amplicons [22,23].

### Study population

All treatment naive patients, registered in DANHEP with chronic HCV infection, who started combination therapy with peginterferon and ribavirin between 1 January 2002 and 1 January 2007, were included in the study (table 1). Patients were included regardless of whether they had completed treatment or not, even if they had only received one dosage of peginterferon. Patients with positive HBsAg or positive anti-HIV antibody test were excluded. In addition, 63 patients were excluded because HCV-RNA status test at treatment initiation was not available.

**Table 1:** Baseline characteristics of 432 patients chronically infected with HCV, treated with peginterferon and ribavirin in Denmark between 1 January 2002 and 1 January 2007

		Whole cohort	
		***SVR***	***Non-SVR***

***Total***		274	158

***Gender***	**Male**	177 (64.5)	105 (66.5)

***Nationality***	**European**	226 (82.5)	146 (92.4)

	**Non-European**	48 (17.5)	12 (7.6)

***Route of infection***	**IDU**	145 (52.9)	74 (46.8)

	**non-IDU**	37 (13.5)	30 (19.0)

	**Unknown**	92 (33.6)	54 (34.2)

***Age at treatment initiation***	**≤ 45 years**	148 (54.0)	49 (31.0)

***Genotype***	**1**	59 (21.5)	74 (46.8)

	**2**	37 (13.5)	13 (8.2)

	**3**	168 (61.3)	67 (42.4)

	**4**	6 (2.2)	4 (2.5)

	**6**	2 (0.7)	0

	**Unknown**	2 (0.7)	0

***HCV-RNA***	**> 600.000 IU/mL**	92 (33.6)	77 (48.7)

***Elevated ALT***	2 × UNL*	149 (54.4)	73 (46.2)

***Treating*** ***Department (speciality)***	**Infectious Diseases**	177 (64.6)	101 (63.9)

	**Gastroenterology/Hepatology**	97 (35.4)	57 (36.1)

***Liver biopsy within 3 years***	**Yes**	152 (55.5)	98 (62.0)

***Treatment completion***	**As planned**	193 (70.4)	65 (41.1)

	**With dose reduction**	55 (20.1)	43 (27.2)

	**Ended before scheduled**	24 (8.8)	39 (24.7)

	**Unknown**	2 (0.7)	11 (7.0)

Data according to those achieving sustained virological response (SVR) versus non-SVR.

Numbers in parenthesis is % of persons with SVR and non-SVR respectively.

UNL, upper normal limit

*2 × UNL = ALT > 70 IU/L (female), ALT > 100 IU/L (male)

IDU, Intravenous drug use

Treatment with either pegylated interferon alpha-2a (Pegasys^®^, Roche A/S, Basel, Switzerland) or alpha-2b (PegIntron^®^, Schering-Plough, Kenilworth, NJ, USA) was given weekly according to the manufacturer's instructions. Ribavirin (Copegus^®^, Roche or Rebetol^®^, Schering-Plough) was given daily, adjusted to body weight for genotypes 1 and 4 or as "flat dosage" for genotypes 2 and 3, according to the manufacturer's instructions. The duration of therapy was planned for 48 weeks for genotype 1 and 4 and 24 weeks for genotype 2 and 3. Some patients with genotype 2 or 3 who were included in a clinical trial were randomized to 12 weeks of treatment [9]. Other genotype 2 or 3 infected patients participating in another clinical trial were randomized to 14 or 24 weeks of treatment, if they had a negative HCV-RNA after 4 weeks [8]. In general, if HCV-RNA titers had not decreased by a minimum of 2 logs after 12 weeks of treatment, treatment was stopped according to international guidelines.

### Study outcome

Treatment outcome was evaluated 24 weeks after end of treatment, where patients with undetectable HCV-RNA were categorized as responders, having achieved sustained virological response (SVR). Patients with detectable HCV-RNA levels 24 weeks after end of treatment were categorized as non-responders.

### Statistics

Logistic regression analyses were used to evaluate potential predictors of SVR, estimating odds ratios (OR) and 95% confidence intervals (CI). We calculated unadjusted and adjusted OR with the following covariates included in the model: Gender, nationality (European vs. Non-European, route of infection (Intravenous drug use (IDU) vs. non-IDU), age at treatment initiation (≤ 45 years vs. > 45 years), HCV genotype (genotype 1 vs. type 2 and 3), ALT at treatment initiation (≤ 2 × UNL vs. > 2 × UNL), HCV-RNA at treatment initiation (≤ 600.000 IU/mL vs. > 600.000 IU/mL), and type of hospital department (Infectious Diseases vs. Gastroenterology/Hepatology). We also adjusted for treatment completion (as scheduled vs. with dose reduction vs. ended before scheduled) (table 2). For those who had a liver biopsy performed within 3 years prior to treatment initiation, we made sub analyses adding the result of the biopsy to the model; cirrhosis vs. not cirrhosis (METAVIR score [24]).

**Table 2:** Unadjusted and adjusted odds ratios and 95% confidence intervals (CI) for sustained virological response after treatment with peginterferon and ribavirin in 432 chronically HCV infected patients, according to predictor variables

	Predictor variable	Odds ratio (95% CI) unadjusted	Odds ratio (95% CI) adjusted
***Gender***	**Female**	1	1

	**Male**	0.92 (0.61-1.39)	0.80 (0.50-1.27)

***Nationality***	**European**	1	1

	**Non-European**	2.53 (1.23-5.21)	3.03 (1.20-7.67)

***Route of infection***	**Non-IDU**	1	1

	**IDU**	1.59 (0.91-2.77)	1.43 (0.76-2.69)

	**Unknown**	1.38 (0.77-2.49)	1.21 (0.61-2.41)

***Age***	**Age ≤ 45 years at treatment initiation**	1	1

	**Age > 45 years at treatment initiation**	0.38 (0.25-0.58)	0.47 (0.30-0.74)

***Genotype***	**1**	1	1

	**2 or 3**	3.01 (1.97-4.60)	2.31 (1.45-3.69)

***HCV-RNA***	**≤ 600.000 IU/mL**	1	1

	**> 600.000 IU/mL**	0.53 (0.36-0.79)	0.70 (0.45-1.10)

***ALT***	Less than 2 × UNL*	1	1

	**More than 2 × UNL**	1.39 (0.94-2.06)	1.40 (0.91-2.16)

***Treating department (speciality)***	**Gastroenterology/Hepatology**	1	1

	**Infectious Diseases**	1.03 (0.69-1.55))	0.93 (0.59-1.49)

***Liver biopsy***	**Not cirrhosis**	1	

	**Cirrhosis**	0.44 (0.25-0.79) ***^§^***	

***Treatment completion***	**As shceduled**	1	1

	**With dose reduction**	0.55 (0.34-0.90)	0.57 (0.33-0.96)

	**Ended before scheduled**	0.22 (0.12-0.38)	0.26 (0.14-0.47)

UNL, upper normal limit

*2 × UNL = ALT > 70 IU/L (female), ALT > 100 IU/L (male)

^§^Only 250 of the 432 patients had a liver biopsy done. Thisparameter is therefore not included in the adjusted analyses.

IDU, Intravenous drug use

Previous studies have shown that patients infected with genotype 2/3 have a higher response rate to combination therapy, and we therefore performed the analysis stratified on HCV genotype (genotype 1 vs. genotype 2/3). Because only 10 and 2 patients were infected with HCV genotype 4 and 6, respectively, these patients were not included in the analyses stratified on HCV genotype.

Starting treatment at a younger age has been associated with higher SVR rates [1,6,8,9,25]. Some studies have included age as a dichotomized variable, while others have included age in the models as a continuous variable. We divided the cohort according to age in 10 groups of equal size and made logistic regression analyses to estimate beta values, to evaluate whether to use a continuous age scale or a specific cut-off point and if so, to estimate the best cut-off point. We observed a sharp drop in beta values between the ages of 43-47, and therefore age was modeled in the regression analysis as equal to or less than 45 vs. over 45 years of age at start of treatment.

Data analyses were performed using SPSS statistical analysis version 16.0 (SPSS, Chicago, Illinois, USA). The Study was approved by the Danish Data Protection Agency (J. nr. 2007-41-0815).

## Results

### Study population characteristics

In total, 432 chronically infected HCV patients were included in the study (table 1). Among the 372 patients of European origin, 327 (87.9%) were Danish. The group of non-Europeans consisted of 10 African-, 3 American-, 9 Asian-, 15 Pakistani-, and 15 Middle Eastern patients. Eight patients had an unknown country of origin. Median age at treatment initiation was 46 years, (range 20-71). Twenty-eight patients (6.5%) received lower start doses of peginterferon, ribavirin, or both, than recommended by the manufacturer.

Genotyping revealed that 133 (30.8%) patients were infected with genotype 1, 50 (11.6%) with genotype 2, 235 (54.4%) with genotype 3, 10 (2.3%) with genotype 4, and 2 patients with genotype 6 (0.5%). In 2 patients (0.5%) the HCV genotype was unknown.

### Effectiveness of treatment with pegylated interferon and ribavirin and predictors of SVR

The overall SVR rate was 63%, with rates of 44%, 74%, 71% and 60% for patients with genotypes 1, 2, 3 and 4, respectively (table 1). Both patients with genotype 6 infection obtained SVR.

**Figure 1:**
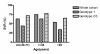
**Sustained virological response (SVR) as a function of age, at antiviral treatment initiation in patients with chronic hepatitis C**. The figure illustrates SVR in the total cohort and SVR depending on genotype, in different age groups. In all, 274 of 432 achieved SVR. 197 patients were ≤ 45 years (148 achieved SVR) and 235 patients were > 45 years (126 achieved SVR). Of the 133 patients with genotype 1 infection, 47 were ≤ 45 years (30 achieved SVR), and of the 285 genotype 2/3 infected patients, 144 were ≤ 45 years (113 achieved SVR).

Genotype, age and nationality all had substantial impact on the fraction of patients who obtained SVR (table 2). Patients infected with genotype 2/3 had an OR of 2.3 for SVR compared to genotype 1 and patients who initiated treatment at age of ≤ 45 years had an OR of 2.1 for SVR. The impact of age at treatment initiation was seen for genotype 1 as well as 2/3, although it was not statistically significant for the latter group (table 3). Figure 1 shows SVR rates according to age. When analyzing only the group of patients ≤ 45 years, genotype was still a predictor of SVR, though not statistically significant, while it was a significant predictor of SVR in the group > 45 years at treatment initiation (table 4). Also the group of non-Europeans had a significantly higher SVR rate with an OR of 3.03 for SVR.

**Table 3:** Odds ratios and 95% confidence intervals (CI) for age as predictor of sustained virological response after treatment with peginterferon and ribavirin in 418 chronically HCV infected patients, divided by genotype (133 genotype 1, 285 genotype 2/3)

		Genotype 1	Genotype 2/3
		Odds ratio (95% CI)*	Odds ratio (95% CI)*

***Age***	**≤ 45 years**	1	1

	**> 45 years**	0.23 (0.09-0.63)	0.58 (0.33-1.00)

			

*adjusted for gender, nationality, route of infection, HCV-RNA, ALT, treating department (gastroenterology/hepatology vs. infectious diseases), treatment completion as scheduled, ended before scheduled or dose reduction

**Table 4:** Odds ratios and 95% confidence intervals (CI) for genotype as predictor of sustained virological response after treatment with peginterferon and ribavirin in 432 chronically HCV infected patients, divided by age

		Age ≤ 45 years	Age > 45 years
		Odds ratio (95% CI)*	Odds ratio (95% CI)*

***Genotype***	**1**	1	1

	**2/3**	1.42 (0.62-3.24)	3.00 (1.65-5.47)

*adjusted for gender, nationality, route of infection, HCV-RNA, ALT, treating department (gastroenterology/hepatology vs. infectious diseases), treatment completion as scheduled, ended before scheduled or dose reduction

In an analysis only including patients who had had a liver biopsy performed prior to treatment initiation, we found that cirrhosis was negatively associated with SVR, but the effect became statistically insignificant after adjustment of other co-factors (table 2).

Data available after treatment initiation showed that dose reduction or stopping treatment before scheduled were strongly associated with a reduced chance of SVR (table 2).

## Discussion

In a nationwide population based cohort of chronically infected HCV patients we observed sustained virological response (SVR) rates of 44% and 72% for genotype 1 and 2/3 respectively, after combination therapy with peginterferon and ribavirin. Other main predictors of SVR were age ≤ 45 years at treatment initiation and completion of non-modified treatment regimens. Finally, being of Non-European nationality predicted a higher SVR rate.

While several controlled clinical trials have evaluated effectiveness of treatment with peginterferon and ribavirin in HCV infected patients, the effectiveness of treatment in a routine clinical setting is less well shown [10,25], except for one retrospective, observational cohort study by Backus et al. with SVR rates of 20%, 52% and 43% in genotypes 1, 2 and 3, respectively, although the patients included in this study were mainly men [12]. However, the present study demonstrated that the effectiveness of treatment, with SVR rates of 44-72%, is comparable to the SVR rates reported in controlled, clinical trials of genotype 1-3 patients [1,2]. The unique perspective of the present study is the nationwide, population based cohort design and a relatively large number of genotype 1, 2 and 3 infected patients included with an almost even distribution of males and females. As only very few patients in Denmark are treated at medical departments not participating in the DANHEP study, our study includes the vast majority of patients treated for HCV in Denmark during the study period.

In accordance with observations obtained in controlled, clinical trials we found both genotype and younger age to be predictors of SVR in patients treated with peginterferon and ribavirin [1,5,6,25], but to our knowledge the present study is the only one, based on a nationwide cohort of unselected patients evenly distributed between males and females, treated in a routine clinical setting, thus representing an important confirmation of the effectiveness of treatment with pegylated interferon and ribavirin observed in controlled, clinical trials. In the present study, infection with genotype 2/3 was a strong predictor of SVR, also in patients ≤ 45 years, though statistical significance was not shown in this group.

In a study by Elefsiniotis et al. [10], genotype was not found as a predictor of SVR in patients younger than 35 years, and age was not a significant predictor of SVR in the overall cohort. However, in their analysis, age was used as a continuous variable. We have not been able to determine why certain cut off points for age have been chosen in other studies evaluating age as a predictive factor of SVR. In the present study, we evaluated whether or not age was best modeled as a continuous variable, or as a design variable dividing age in two groups. We found that the age of 45 was a relevant cut off point.

Even though Antonucci et al. [25] only included half as many patients with genotype 1 and a third as many with genotype 2/3, as we did, in accordance with our results, they found that age of < 40 years was a significant predictor of SVR in genotype 1 infected patients treated outside clinical trials. Similarly to Antonucci et al, we did not find younger age to be a statistically significant predictor of SVR in genotype 2/3 infected patients. In our study however, there was a trend towards younger age also being a predictor of SVR in genotype 2/3 (OR: 0.58, 95% CI: 0.33-1.00). Dalgard et al. [8] and Lagging et al. [9], included 428 and 382 highly selected genotype 2/3 patients, respectively, and found that younger age predicted a higher chance of SVR. Older age at the time of infection is known to be associated with development of liver fibrosis [26-28]. Patients are carefully selected before initiation of antiviral treatment, because the overall treatment outcome is "poor", and treatment is lengthy, with many potentially severe side effects. International guidelines on whom to treat suggest that there should be evidence of liver fibrosis before treatment initiation in genotype 1 patients [29,30]. Due to the better treatment outcome and shorter treatment duration, patients infected with genotype 2 or 3 can start treatment without prior liver biopsy.

A former study, based on data from DANHEP [19], showed that patients infected with genotype 2/3, had an approximately 2.5 times higher chance of starting antiviral treatment, while older age at referral was a negative predictor of treatment initiation. The results in the present study, with relatively high SVR rates in genotype 1 patients age 45 or younger, could have implications for future treatment strategy in this group of patients. Whether younger patients undergoing the new upcoming treatment regimens that add a specific HCV protease inhibitor to the standard of care treatment, can benefit from even shorter treatment periods remains to be seen. Clearly, further studies are required to establish the specific indications for treatment initiation in younger patients. Being infected with HCV has been shown to decrease quality of life, regardless of disease progression, and SVR after treatment improves quality of life [31]. This is another aspect that should be considered when deciding whether or not to treat patients for chronic hepatitis C.

Recently, a genetic variation near the IL28B gene on chromosome 19 was found to be significantly associated with SVR in HCV infected genotype 1 patients, treated with peginterferon and ribavirin [32-34]. Ge et al. [32] found this advantageous genetic variation in 35% of the cohort examined. Patients with this variation had a 2-3 fold higher rate of SVR than other patients. Around 25-40% of the patients without the advantageous genetic variation gained SVR, and the genetic variation was only associated with an SVR in patients who did not become HCV RNA negative at week 4 in patients with genotypes 2 and 3 [35]. Therefore it is still important to identify and use other predictors of SVR when deciding whether to start treatment for HCV infection. Genetics do not change with age, therefore the main findings of the present study are still very important. It is possible that patients with genotype 1 HCV infection without the advantageous IL28B genetic variation, identified in the studies mentioned above, could benefit from treatment before the age of 45 years.

The association between being of non-European origin and the high SVR rate could not be explained from our data. Thus, further research is needed to elucidate whether this difference could potentially be related to IL28B polymorphism differences, as the group of non-Europeans was diverse ethnically and data on IL28B-polymorphisms do not exist for all ethnicities involved in this study.

There are some limitations to the present study. The study is a retrospective observational study, and we are lacking information on some potentially important confounders such as weight, prior depression, and prior raised alcohol intake. We did not have any information on viral kinetics during treatment, and therefore we could not include this factor in the analyses. Furthermore, we did not have any knowledge of which patients were treated in clinical trials. It could have been interesting to determine if being treated in a clinical trial was a predictor of SVR.

## Conclusions

We conclude that in a routine clinical practice the treatment effectiveness of patients with chronic HCV with peginterferon and ribavirin is equivalent to the effectiveness observed in clinical trials. Furthermore, genotype 2 and 3, age ≤ 45 years at treatment initiation and completion of a non-modified treatment regimen is strongly associated with a sustained treatment response. Thus, our study on a nationwide Danish cohort shed new light on combination therapy of chronic HCV.

### Potential conflict of interest

Peer Brehm Christensen is member of the Advisory Board for Roche A/S and has received research grants from Roche A/S and Sheering Plough.

Nanna Hansen; No conflict, Niels Obel; No conflict, Mette Kjær; No conflict, Alex Lund Laursen; No conflict, Henrik Bygum Krarup; No conflict, Axel Møller; No conflict, Poul Schlichting; No conflict, Jens Bukh; No conflict, Nina Weis; No conflict.

No authors listed in appendix 1 have any conflict of interest

## Authors' contributions

NH, NO, JB, NW have made substantial contributions to conception and design, analysis and interpretation of data and drafting the manuscript; NH, NO, PBC, MK, ALL, HBK, AM, PS, JB, NW have made substantial contributions to acquisition of data and all authors contributed with a critical revision of the manuscript and read and approved the final manuscript.

## Pre-publication history

The pre-publication history for this paper can be accessed here:

http://www.biomedcentral.com/1471-2334/11/177/prepub
